# The Fate of Autologous Endometrial Mesenchymal Stromal Cells After Application in the Healthy Equine Uterus

**DOI:** 10.1089/scd.2018.0056

**Published:** 2018-08-01

**Authors:** B. Elisabeth Rink, Teresa Beyer, Hilari M. French, Elaine Watson, Christine Aurich, F. Xavier Donadeu

**Affiliations:** ^1^ Department of Clinical Sciences, Ross University School of Veterinary Medicine, St.Kitts, West Indies.; ^2^ The Roslin Institute, University of Edinburgh, Edinburgh, United Kingdom.; ^3^ Artificial Insemination and Embryo Transfer, Department for Small Animals and Horses, University of Veterinary Medicine, Vienna, Austria.

**Keywords:** mesenchymal stem cells, equine, endometrial, uterine

## Abstract

Because of their distinct differentiation, immunomodulatory, and migratory capacities, endometrial mesenchymal stromal cells (MSCs) may provide an optimum source of therapeutic cells not only in relation to the uterus but also for regeneration of other tissues. This study reports the fate of endometrial MSCs following intrauterine application in mares. Stromal cell fractions were isolated from endometrial biopsies taken from seven reproductively healthy mares, expanded, and fluorescence labeled in culture. Phosphate-buffered saline (PBS) or MSCs (15 × 10^6^) were autologously infused into each uterine horn during early diestrus and subsequently tracked by fluorescence microscopy and flow cytometry of endometrial biopsies and blood samples taken periodically after infusion. The inflammatory response to cell infusion was monitored in endometrial cytology samples. MSCs were detected in endometrial sections at 6, 12, and 24 h, but not later (7 or 14 days), after cell infusion. Cells were in all cases located in the uterine lumen, never within the endometrial tissue. No fluorescence signal was detected in blood samples at any time point after infusion. Cytology analyses showed an increase in % of polymorphonuclear neutrophils between 1 and 3 h after uterine infusion with either MSCs or PBS and a further increase by 6 h only in mares infused with PBS. In summary, endometrial MSCs were detected in the uterine lumen for up to 24 h after infusion, but did not migrate into the healthy endometrium. Moreover, MSCs effectively attenuated the inflammatory response to uterine infusion. We conclude that endometrial MSCs obtained from routine uterine biopsies could provide a safe and effective cell source for treatment of inflammatory conditions of the uterus and potentially other tissues.

## Introduction

Tissue regeneration approaches based on stem cells have been clinically used in horses for over 15 years, primarily involving the use of mesenchymal stromal cells (MSCs) obtained from bone marrow (BM) and adipose tissue (AT) [1–4] to enhance recovery of tendons, ligaments [5–7], or diseased joints [8,9]. Although not widely explored, the therapeutic potential of MSCs in veterinary species extends well beyond orthopedic applications, as clearly shown in the human field [10–12].

The endometrium has recently emerged as a promising alternative source of therapeutic MSCs. Endometrial tissue can be easily obtained by biopsy without the use of anesthesia, in contrast to the relatively invasive procedures of BM aspiration or liposuction more traditionally employed to obtain MSCs; in the case of humans, menstrual blood constitutes an even less invasive source of MSCs [13,14]. Endometrial MSCs were first described in humans [15,16] and were later isolated and characterized from mice [17,18] and several domestic species [19–25]. More recently, we were the first to identify, isolate, and successfully expand in vitro cells from the equine endometrium that fulfill the definition of MSCs [26]. These cells exhibit a high proliferation rate; robustly express typical MSC markers such as CD29, CD44, CD90, and CD105, but lack expression of the hematopoietic markers, CD34 and CD45; and differentiate into adipogenic, osteogenic, and chondrogenic lineages. Additionally, compared with BM MSCs, endometrial MSCs have a distinct ability to undergo smooth muscle differentiation [26] and display robust immunomodulatory [27] and migratory [28] properties.

MSC infusion has been proposed for the treatment of highly prevalent endometrial conditions in mares, including persistent mating-induced endometritis and endometrosis, a condition not to be confused with endometriosis in humans, but leading to uterine gland degeneration and infertility [29–31]. Ferris et al. showed that BM-derived MSCs infused into the uterus 24 h before insemination were able to modulate the uterine inflammatory response to spermatozoa in healthy mares. In 2013, Mambelli and associates reported that AT MSCs infused into the uterine lumen effectively migrated and engrafted in periglandular spaces of equine endometria [30]. The group furthermore reported improvement of histological changes associated with endometrosis after uterine infusion and concluded that multiple mechanisms, including homing to fibrotic periglandular and glandular spaces, and increased epithelial cell proliferation might mediate the antiscarring effects of AT MSCs [32]. Moreover, Falomo et al. found that AT MSCs could infiltrate the periglandular space as well as single glands of endometrosis-affected tissue in vitro [33].

We reasoned that because of their origin as well as distinct differentiation, immunomodulatory, and migratory capacities, endometrial MSCs may provide an optimum source of therapeutic cells for uterine applications. With this in mind, the present study aimed to follow the fate of endometrial MSCs (obtained from routine endometrial biopsies) after autologous transplantation into the healthy uterus during diestrus, with the hypothesis that transplanted cells would be able to effectively infiltrate the endometrium. Additionally, we aimed to monitor the inflammatory reaction of the endometrium to intrauterine cell infusion.

## Materials and Methods

### Experimental mares and design

The study was performed according to the Austrian animal welfare legislation and was approved by the competent authority for animal experimentation (Austrian Federal Ministry for Science and Research, license numbers BMWFW-68.205/0069-WF/3b/2016 and BMWFW-68.205/0103-WF/V/3b/2017).

A total of seven cycling mares of the Haflinger breed, aged 4–7 years (mean 5.3 years), belonging to the Centre of Artificial Insemination and Embryo Transfer, Vienna, Austria, were included in the study. The mares were housed in outdoor paddocks and kept in their established group. They were fed hay twice daily and had ad libitum access to water. Mares were checked for signs of estrus thrice a week by ultrasound examination. When in estrus, defined by a follicle >3.5 cm and pronounced uterine edema, mares were checked daily to determine the day of ovulation. A breeding soundness examination was performed before the beginning of the study to confirm reproductive health, which included a transrectal ultrasonography examination, a bacterial swab, and staging of tissue from an endometrial biopsy according to Kenney and Doig [34]. All mares were free of uterine fluid and had a negative uterine bacterial culture and a biopsy grade I to IIa.

**FIG. 1.**
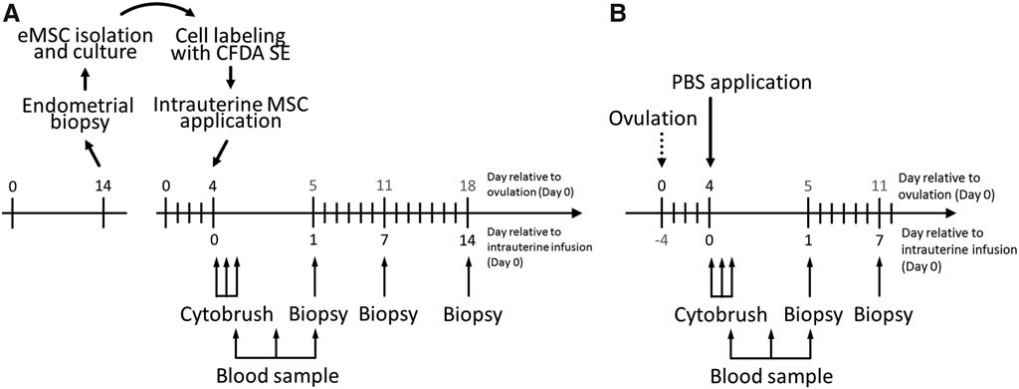
Experimental design. **(A)** MSCs were harvested through endometrial biopsy from 7 mares on day 14 of the cycle before intrauterine infusion and were expanded in culture and labeled with CFDA SE. MSCs were then infused autologously into the uterus on day 4 of the cycle, followed by collection of endometrial cytology samples at 1, 3, and 6 h, blood samples at 6, 12, and 24 h, and endometrial biopsies at 24 h and 7 and 14 days after cell infusion. **(B)** Control mares (*n* = 4) received intrauterine PBS on day 4 of the cycle. The same follow-up sampling protocol was used as for the MSC infusion group except that only two biopsies were collected. MSCs, mesenchymal stromal cells; PBS, phosphate-buffered saline.

An experimental design was used as shown in Fig. 1A whereby MSCs were obtained from endometrial biopsies collected from a total of 7 mares on day 14 after ovulation. After expansion in culture, MSCs were infused autologously into the uterus during the next cycle (day 4 after ovulation), followed by periodic uterine and blood sampling. In addition, four of these mares were also used as controls in which, two estrous cycles before the MSC infusion cycle, they received intrauterine phosphate-buffered saline (PBS) (1 mL) on day 4 after ovulation, followed by the same sampling protocol used after MSC infusion, except that a third biopsy was not collected (Fig. 1B).

### Experimental procedures

#### Harvest and culture of MSCs

All chemicals and reagents used for cell culture were obtained from Life Technologies (Thermo Fisher Scientific, Waltham, MA) unless otherwise specified, and Nunc^™^ culture plasticware was purchased from Sigma Aldrich (St. Louis, MO).

For all intrauterine procedures, mares were restrained in an examination stock, the tail was wrapped, and the perineum and vulva were washed with disinfectant soap, rinsed with clean water three times, and dried with paper towels. All intrauterine procedures were performed with sterilized equipment and the examiner wearing long, sterile obstetric gloves to minimize the potential of uterine contamination.

Before taking biopsies, transrectal ultrasonography was performed on each mare to confirm the presence of a corpus luteum and absence of intrauterine fluid. Two endometrial biopsy specimens were collected with a standard alligator-type biopsy punch [35] and were immersed in 50 mL of PBS on ice. Biopsy specimens were immediately processed for MSC isolation and culture, as described [26]. In short, endometrial tissue was minced and incubated in dissociation medium (phenol red-free DMEM/F-12 containing 0.1%BSA, 0.5% collagenase I, 40 μg/mL deoxyribonuclease type I, and 1% penicillin/streptomycin) for 40 min at 37°C, rotating at 150 rpm in a laboratory shaker (Labnet, Orbit 1000) placed in an incubation hood (Unihood 550). The resulting cell solution was filtered through a sterile 70-μm cell strainer (Fisher Scientific) to eliminate undigested tissue fragments. Thereafter, cells were washed in culture medium (phenol red-free DMEM/F-12 containing 10% fetal bovine serum (FBS) and 1% penicillin/streptomycin) and resuspended in Ca2+ and Mg2+-free PBS supplemented with 0.1% FBS and 2 mM sodium citrate. Magnetic Dynabeads M-450 (Thermo Fisher Scientific) coated with a mucin-1 (Muc-1) antibody (Santa Cruz, Dallas, TX), according to the manufacturers' protocol, were utilized to remove epithelial cells. Samples were incubated with beads for 40 min at 4°C with gentle rotation and tilting. The unbound (Muc-1-negative) stromal cell fraction was collected using a Dynamagnet 2 (Thermo Fisher Scientific), washed, resuspended in culture medium, and cultured in a humidified incubator at 37°C in 5% CO_2_:95% air. Culture medium was changed every 2–3 days, and MSCs were passaged using TrypLE when 80% confluent.

#### Intrauterine cell infusion

After trypsinization, cells were labeled with the Vybrant CFDA SE Cell Tracer Kit (Invitrogen, Thermo Fisher Scientific) as per the manufacturer's instructions. The viability of labeled cells was estimated with Trypan blue before cells were resuspended in 1 mL of PBS for intrauterine infusion. The mares were prepared as described before and a flexible disposable insemination pipette (Minitube, Tiefenbach, Germany) was introduced into the vagina. The insemination pipette was inserted into the uterus through the cervix and guided into the left uterine horn first, assisted by transrectal palpation. Approximately 15 million MSCs were released into the left uterine horn using a 0.5-mL embryo transfer straw and flexible stylet normally used for embryo transfer procedures. The pipette was slightly retracted and subsequently placed into the right uterine horn where another 0.5-mL straw was used to deposit 15 million MSCs.

#### Uterine cytology

Samples were collected using a double-guarded cytology brush, as described [36]. Brushes were subsequently rolled onto each of the three glass microscope slides, allowed to dry, fixed, stained with a commercially available Diff Quick stain LT-SYS^®^ (Labor + Technik, Berlin, Germany), and finally mounted. Cytology smears were later visualized and examined under a Zeiss Imager.Z2 microscope. Five fields of vision at 200-fold magnification were examined, cells counted, and the percentage of polymorphonuclear neutrophils (PMNs) relative to epithelial cells (indicated as % PMNs) was calculated using the following formula: $\% \;PMN = { \frac { PMN }  { epithelial \;cells } } \times \;100$. Inflammation was graded as nonexistent (<5% neutrophils), mild (5%–10% neutrophils), moderate (15%–30% neutrophils), and severe (>30% neutrophils) according to Card [37].

#### Blood sampling and flow cytometry

Blood samples were collected from the jugular vein into EDTA tubes. Samples were diluted 1:1 in prewarmed PBS and 4 mL of this solution was underlaid with 2 mL of Ficoll-Paque (GE Healthcare, Uppsala, Sweden) and centrifuged at room temperature for 40 min at 400 g with brakes switched off. Mononucleated cells were collected from the Ficoll-Paque–medium interface, washed, and resuspended in PBS. The dead cell stain Cytox blue (Life technologies, Fisher Scientific) was used according to the manufacturer's protocol and cells were analyzed using a BD FACS Canto II flow cytometer equipped with FACS Diva software. A total of 2.5 × 10^6^ events were recorded and data were analyzed with FlowJo (version 10). CFDA SE-labeled cells were analyzed as positive controls.

#### Immunofluorescence

Follow-up biopsies were collected from the base of the right uterine horn, snap-frozen immediately, and later cut into 5-μm sections using a CryoStar NX70 (Thermo Scientific) cryotome. Sections were visualized under a Zeiss Imager.Z2 fluorescence microscope.

### Statistical analysis

Results are shown as mean ± standard error. Normal distribution of data was confirmed using the Kolmogorov–Smirnov test and data were analyzed by two-way analysis of variance followed by Tukey's test using Minitab statistics software (Minitab, Inc., State College, PA). Significance was set at *P* < 0.05.

## Results

From each mare, tissues from two endometrial biopsies were digested, yielding an average of 10 million cells, which were then processed by magnetic beading. Culture of the resulting unbound (stromal) fraction for ∼2 weeks yielded a minimum of 30 million cells, which were used for autologous application.

No accumulation of uterine fluid was detected in any mare during the study. Mild accumulation of air was detected in most mares after uterine manipulation, but this had resolved by 24 h.

### 

#### Flow cytometry analyses of MSCs in peripheral blood

No fluorescence signal could be detected in blood samples from either MSC- or PBS-infused mares at 6, 12, and 24 h after application (Supplementary Fig. S1; Supplementary Data are available online at www.liebertpub.com/scd).

#### Tracking of MSCs in endometrial biopsies

**FIG. 2.**
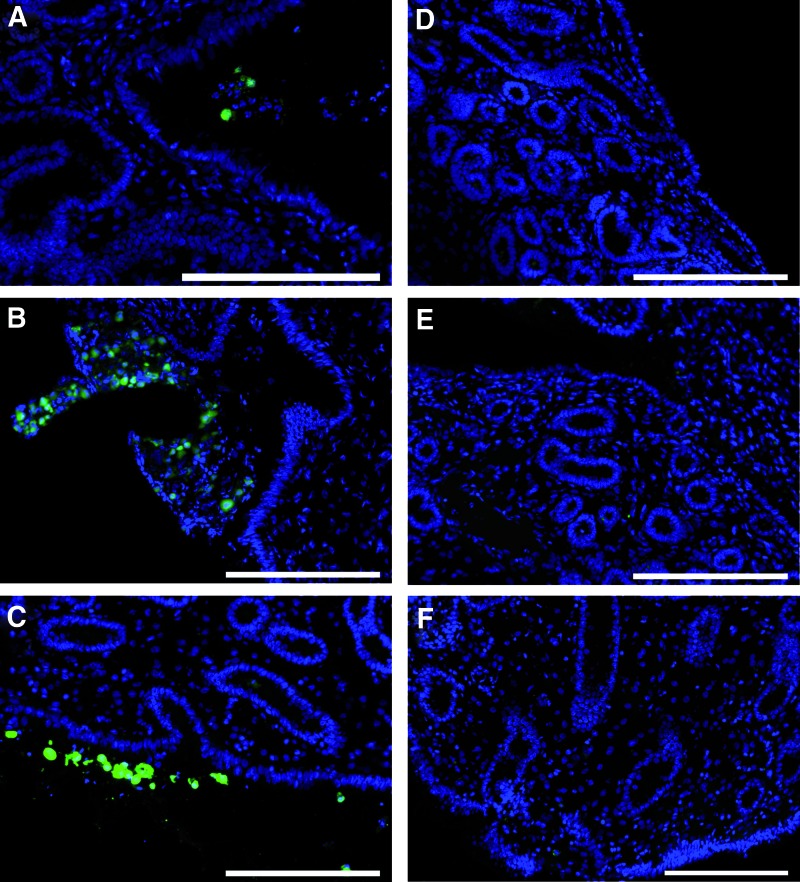
Representative images showing localization of CFDA SE-labeled endometrial MSCs in sections collected at **(A)** 6, **(B)** 12, and **(C)** 24 h after infusion. No labeled cells were detected in sections collected at **(D)** 7 and **(E)** 14 days after application. **(F)** Negative control. Scale bars 200 μm. Color images available online at www.liebertpub.com/scd

**FIG. 3.**
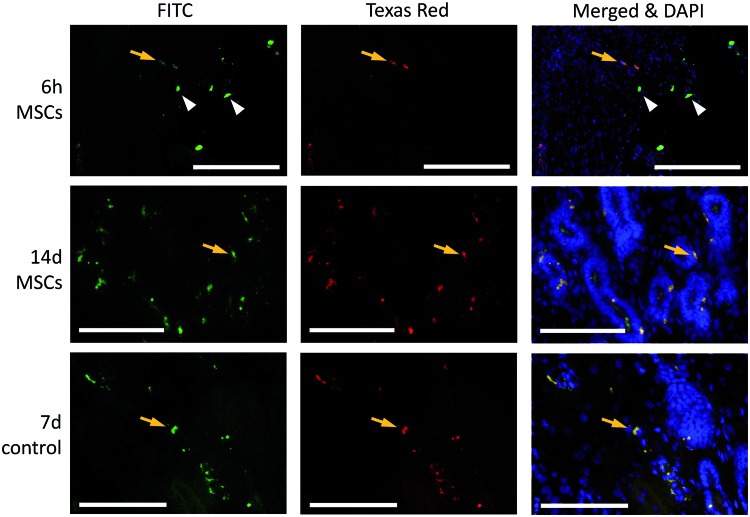
Representative photomicrographs showing autofluorescence (*orange arrows*) in endometrial sections from MSC- and PBS-infused mares at 6 h and 7 and/or 14 days after infusion. CFDA SE-labeled MSCs are indicated by *white arrowheads*. Scale bars at the *lower right* of the image are 200 μm, scale bars at the *lower left* of the image are 100 μm. Color images available online at www.liebertpub.com/scd

We began by collecting biopsies at 24 h and 7 and 14 days after intrauterine infusion in 5 mares. Our analyses in these mares indicated that fluorescence-labeled MSCs could be detected in some of the biopsies collected at 24 h, but not in those collected at 7 or 14 days. Thus, from the two additional mares subsequently infused with MSCs, additional biopsies were taken at 6 and 12 h after infusion. Considering all mares, fluorescence-labeled cells were detected in biopsies collected at 6 h (2/2 mares), 12 h (2/2), and 24 h (4/7, including the two mares collected at 6 and 12 h), but not 7 or 14 days (0/7), after MSC infusion (Fig. 2). MSCs were in all cases located exclusively in the uterine lumen. Moreover, no fluorescence-labeled cells were identified in biopsies collected after infusion with PBS. However, autofluorescence was occasionally detected within the endometrial stroma and close to endometrial glands in sections from either group of mares (Fig. 3).

#### Analyses of PMNs in endometrial cytology samples

**FIG. 4.**
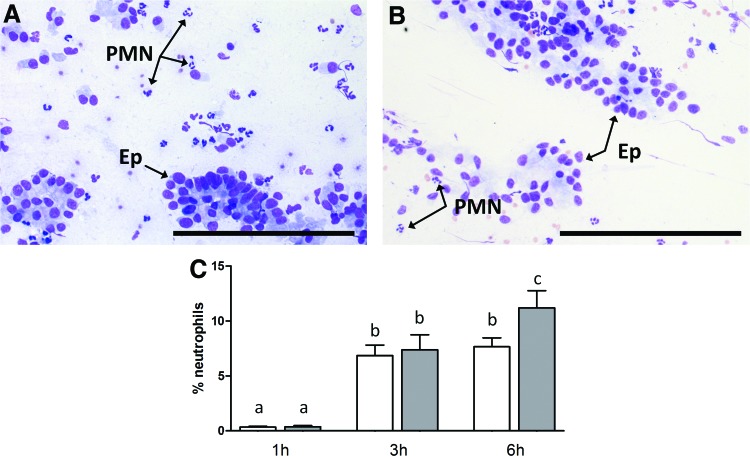
Representative photomicrographs of cytology smears collected from mares infused with **(A)** MSCs (6 h) and **(B)** PBS (3 h). Scale bars **(A, B)** 200 μm; Ep, epithelial cells; PMNs, polymorphonuclear neutrophils. **(C)** Percentages of PMNs at 1, 3, and 6 h after infusion of MSCs (*n* = 7 mares, *white bars*) or PBS (*n* = 4 mares, *gray bars*). *Different superscripts* (a, b, and c) indicate mean differences (*P* < 0.05). Color images available online at www.liebertpub.com/scd

Percentages of neutrophils obtained from cytobrushes are shown in Figure 4. In both MSC- and PBS-infused mares, percentages of PMNs increased from about 0.3% to 7.0% between 1 and 3 h after infusion, and further increased between 3 and 6 h in PBS-infused mares only. The percentage of PMNs was lower in MSC- than PBS-infused mares at 6 h (7.65% ± 0.81% vs. 11.2% ± 1.56%, *P* < 0.02).

## Discussion

This study reports for the first time the isolation of endometrial MSCs from biopsy specimens in mares as well as the fate of these cells after intrauterine infusion. In humans, routine endometrial biopsy and curettage can be used effectively to obtain endometrial MSCs [38–40]. In horses, endometrial biopsies are a safe and routine diagnostic approach and can be collected repeatedly [41]. In this study, we demonstrate that biopsy collection is a suitable method for isolating adequate numbers of endometrial MSCs for subsequent short-term expansion in culture to obtain sufficient cell numbers for intrauterine infusion.

Although CFDA SE-labeled MSCs were detected in the uterine lumen at all time points during the first 24 h after infusion, no MSCs were located within the endometrium. Furthermore, no MSCs could be traced in any of the biopsies collected 7 and 14 days after infusion, overall suggesting that infused endometrial MSCs do not have the capability to migrate through an intact endometrium. In contrast, a previous study found that allogeneic AT MSCs homed in the periglandular area and in single glands of uteri of mares with endometrosis [30]. Thus, pre-existing endometrial pathology with disruption of the epithelial lining is likely to be a prerequisite for MSCs to penetrate the endometrial epithelium and incorporate into periglandular spaces, a conclusion that is consistent with previous results in vitro [33].

Our results indicate that MSCs were being cleared from the uterus by 24 h postinfusion, likely promoted by mild inflammation induced by cervical and uterine manipulation after cell infusion during early diestrus. Yet, we were not able to detect fluorescent cells in blood, consistent with the finding that cells did not migrate through the endometrium. If any cells were indeed able to reach circulation, they may have cleared quickly by entrapment in the lungs, as reported for MSCs applied systemically in other studies [42,43].

Autofluorescence results from natural light emission across a broad excitation and emission spectra by tissue-intrinsic components and/or as a result of tissue processing [44,45]. We clearly detected autofluorescence around blood vessels and from single cells within the endometrial stroma and endometrial glands. This may easily be misinterpreted as migration of MSCs through endometrial stroma. Instead, upon careful examination, it was clear that a true signal from MSCs originated from the densely packed CFDA SE tracer in the cell cytoplasm emitting with an intensity much higher than autofluorescence and specifically in the FITC channel. These observations highlight the importance of accurately identifying fluorescent-labeled cells in histological samples in the course of MSC-tracing studies to avoid false positive results leading to mistaken conclusions.

In mares, an inflammatory reaction occurs physiologically in the uterus following insemination during estrus. Such a response is important in aiding uterine clearance and normally resolves within 24–48 h [46–48]. This inflammatory reaction involves the recruitment of PMNs, which in healthy mares is not apparent at 2 h, but peaks at 6 h after insemination [49]. In our study, MSCs were infused at a relatively low volume (1 mL) and during early diestrus rather than estrus to avoid ready elimination from the uterine lumen. A mild inflammatory reaction occurred shortly (within 3 h) after intrauterine infusion as expected, but was clearly attenuated by the presence of MSCs, as indicated by a lower percentage of PMNs in MSC-infused mares at 6 h. This observation is consistent with results from a previous study in mares, in which allogeneic AT MSCs were infused into the uterus 24 h before insemination [29]. In that study, MSC infusion was associated with an increase in the anti-inflammatory cytokine IL-1Ra and a reduction in proinflammatory IL-1, resulting in decreased neutrophil migration into the uterine lumen in response to insemination. The above effects are consistent with well-described immunomodulatory properties of MSCs [50] and highlight the potential of MSCs for treating postbreeding endometritis in mares.

We conclude that relatively large numbers of MSCs can be obtained after short-term expansion (2 weeks) of cells collected from simple endometrial biopsies in mares. Endometrial MSCs can be safely infused autologously into the uterus during early diestrus where they display immunomodulatory properties. Although MSCs can be detected in the uterine lumen for up to 24 h after infusion, they do not engraft into healthy endometrium. These results highlight the significant potential of the endometrium as a novel source of therapeutic cells for inflammatory and degenerative conditions of the uterus and potentially other tissues.

## Supplementary Material

Supplemental data
