# Supplementary material for: The Fate of Autologous Endometrial Mesenchymal Stromal Cells After Application in the Healthy Equine Uterus
Source: Stem Cells Dev. 2018 Aug 1;27(15):1046–52. doi: 10.1089/scd.2018.0056 (PMC6067096; doi:10.1089/scd.2018.0056)
Supplement: Supplemental data [file Supp_Fig1.pdf]

Supplementary Data

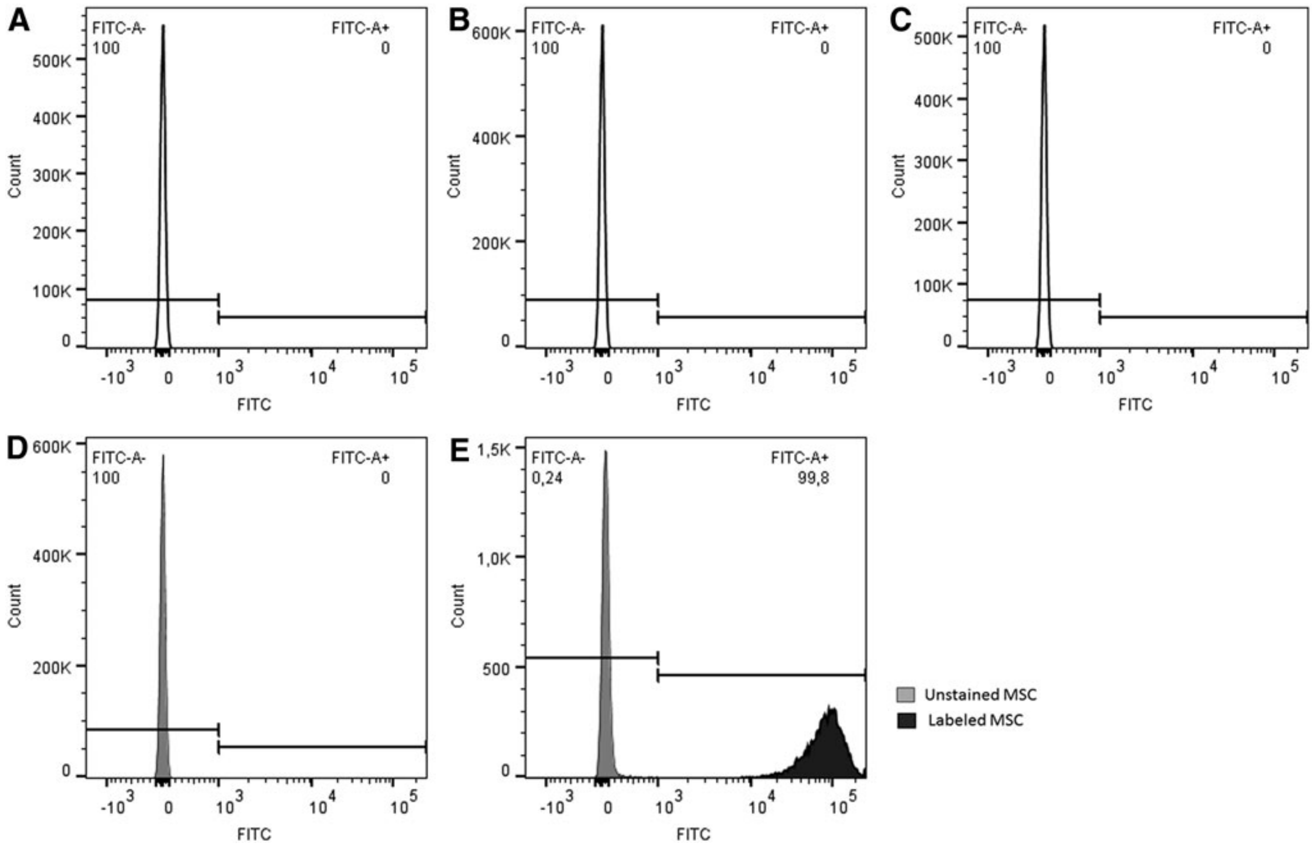

SUPPLEMENTARY FIG. S1. Representative graphs of flow cytometry results. Histograms obtained from flow cytometry analyses of blood samples collected **(A)** 6 h, **(B)** 12 h, and **(C)** 24 h after mares were infused with MSCs or **(D)** 6 h after phosphate-buffered saline application in control mares. CFDA SE-labeled MSCs were used as positive controls **(E)**. $n = 4$ mares/group. MSCs, mesenchymal stromal cells.
